# Imaging findings of neurologic complications in lung transplantation: Review of a 9-year cohort

**DOI:** 10.1177/20584601211038721

**Published:** 2021-10-03

**Authors:** Elena Marín-Díez, Marta Drake-Pérez, Natalia Valle-San Román, Víctor Manuel Mora Cuesta, Miguel Ángel Hernández-Hernández, Enrique Marco de Lucas

**Affiliations:** 1 Department of Radiology, 16516 Marquis of Valdecilla University Hospital, Santander, Spain; 2 Department of Neumology, 16516 Marquis of Valdecilla University Hospital, Santander, Spain; 3 Department of Critical Care Medicine, 16516 Marquis of Valdecilla University Hospital, Santander, Spain

**Keywords:** Lung transplantation, neurological complications, brain imaging, cerebrovascular complications, encephalopathy

## Abstract

**Background:**

Lung transplantation (LT) requires complex multidisciplinary organization and constitutes a therapeutic option and a life-saving procedure. Although the number of lung recipients continues to increase, neurological complications and death rates following lung transplantation are still higher than desirable.

**Purpose:**

This study aims to analyse the neuroimaging findings in a cohort of adult patients with LT.

**Material and Methods:**

A retrospective cohort study of all lung transplant recipients (344 patients: 205 men and 139 women) at a single institution from January 2011 to January 2020. The collected data included demographic features, clinical data and evaluation of the imaging findings. We also recorded the date of neurological complication(s) and the underlying disease motivating lung transplantation.

**Results:**

We found an elevated rate of neuroimaging findings in patients following LT with 32.6% of positive studies. In our cohort, the average time after LT to a neurological complication was 4.9 months post-transplant. Encephalopathy, critical illness polyneuropathy and stroke, in that order, were the most frequent neurological complications. Structural abnormalities in brain imaging were more often detected using MRI than CT for indications of encephalopathy and seizures.

**Conclusions:**

LT recipients constitute an especially vulnerable group that needs close surveillance, mainly during the early post-transplant period.

## Introduction

Lung transplantation (LT) constitutes a therapeutic option that has been escalating since it constitutes a potentially life-saving procedure. Approximately 3500 people worldwide receive a transplant every year.^1–4^ Although the number of lung recipients continues to increase, death rates following lung transplantation are still higher than desirable. Our institution is the fourth largest hospital in our country by number of performed lung transplantations. National 5-year and 10-year survival rates of lung transplant recipients are, respectively, 41.0% and 34.1%.^4^ Neurological complications after LT are common, affecting between 45% and 92% of patients in some studies.^2,3,5^ The influence of nervous system disorders on lung transplant recipients and their survival has been studied. However, reviews of large cohorts, focused on neuroimaging findings in lung transplant recipients, have not been previously published. Therefore, the purpose of this study is to analyse the neuroimaging findings of a complete cohort of adult patients who underwent lung transplantation at our institution over the past 9 years.

## Methods and materials

### Study design and patients

A retrospective cohort study of all lung transplant recipients (344 patients: 205 men and 139 women) at a single institution, from January 2011 to January 2020. Data collection occurred between March 2018 and May 2020. All transplant recipients were carefully followed-up at this institution, setting up regular visits with the lung-transplant team. We subsequently reviewed the existing medical records and diagnostic imaging of all patients who underwent lung transplantation between the period specified. The collected demographic features included sex, age, unilateral or bilateral transplantation and date of transplantation. We also recorded the date of neurological complication(s), the date of death or last follow-up and the underlying disease motivating lung transplantation.

### Imaging techniques and analyses

Imaging was performed on different CT and MRI scanners. Head CT was performed either with or without intravenous contrast medium. MRI brain studies were obtained at 1.5 T (Signa LX General Electric Medical Systems, Waukesha, WI) and 3 T (Achieva 3.0T Philips, Best, The Netherlands) with a standard 8-channel head-coil and with standard protocols, including sagittal T1-weighted, axial T2-weighted, 3D FLAIR sequence, axial diffusion-weighted and intravenously enhanced T1-weighted axial, coronal and sagittal images. The CT scans and MRIs were evaluated by the neuroradiology department staff of our centre. All available images were analysed in a PACS workstation. The imaging data and the diagnostic reports were collected and reviewed by a radiology resident and a senior faculty member with 15 years of neuroimaging experience. Structural abnormalities in brain imaging were classified in the following categories: cerebrovascular complications (including intraparenchymal haemorrhage, subarachnoid haemorrhage and acute/subacute ischaemic infarction), CNS infections, CNS malignancies and drug toxicity with acute/subacute abnormality such as posterior reversible encephalopathy syndrome (PRES). We excluded all the imaging abnormalities associated to chronic and previous changes. All abnormalities were recorded with information on timing (in months after transplantation). Variables were summarized as median (continuous variables) and frequency (ordinal variables).

### Ethical standards

All procedures performed in our study were in accordance with the ethical standards set by our local committee and with the Declaration of Helsinki principles. The informed consent was not needed in this retrospective study.

## Results

### Patient demographics (Tables 1 and 2)

Table 1 summarizes all the information related to the patient demographics of our 9-year cohort. The three most common indications for lung transplantation were interstitial lung disease (42.4%), chronic obstructive pulmonary disease (34.2%) and alpha-1 antitrypsin deficiency (6.6%), although cystic fibrosis was also a common indication in the younger patients. The most frequent entity of the interstitial lung disease group was the usual interstitial pneumonia followed by non-specific interstitial pneumonia. The main causes of occupational lung disease were extrinsic allergic alveolitis, followed by silicosis and asbestosis. In the pulmonary hypertension group, 4 patients had primary pulmonary hypertension and the other 2 had developed pulmonary hypertension due to Takayasu arteritis. In the last group of Table 2 (named Other), we found a variety of entities, mostly quite infrequent disorders.

**Table 1.** Patient demographics.

All patients	
Number of patients	344
Recipient age at transplantation	55.8 yo (20–71)
Recipient gender	
Female	139 (40.4%)
Male	205 (59.6%)
Transplant type	
Unilateral	120 (34.9%)
Bilateral	224 (65.1%)
Number of patients depending on the year of transplantation	
2011	30
2012	43
2013	33
2014	49
2015	36
2016	41
2017	31
2018	41
2019	40

**Table 2.** Indication for LT in our cohort.

	No. of patients
Indication for transplantation	
Interstitial lung disease	144
Chronic obstructive pulmonary	114
disease	22
Alpha-1 antitrypsin deficiency	22
Cystic fibrosis	20
Occupational lung disease	9
Pulmonary hypertension	2
Sarcoidosis	1
Lung malignancy	6
Other	4

### Total of neurological events and brain imaging studies (Figure 1)

In the 9-year study period, 125 patients had neurological manifestations (36.3% of the 344 total lung transplants performed) and 89 patients (71.2% of the 125 recipients with neurological manifestations) had brain imaging studies. Ninety patients had one neurological complication, 31 had two neurological complications and 4 had three neurological complications (28.0% of the patients with symptoms had two or more complications). Twenty-nine patients (32.6% of the imaged patients) had structural abnormalities in brain imaging. The average time to a complication in our cohort was 4.9 months. CT was frequently the first and only imaging technique performed in many of our patients, and only 20 MR studies could be completed. This is due to the severe situation of these patients that limits their access and collaboration to obtain an MR study in daily practice (long acquisition, uncollaborative patients, etc.).

**Figure 1.**
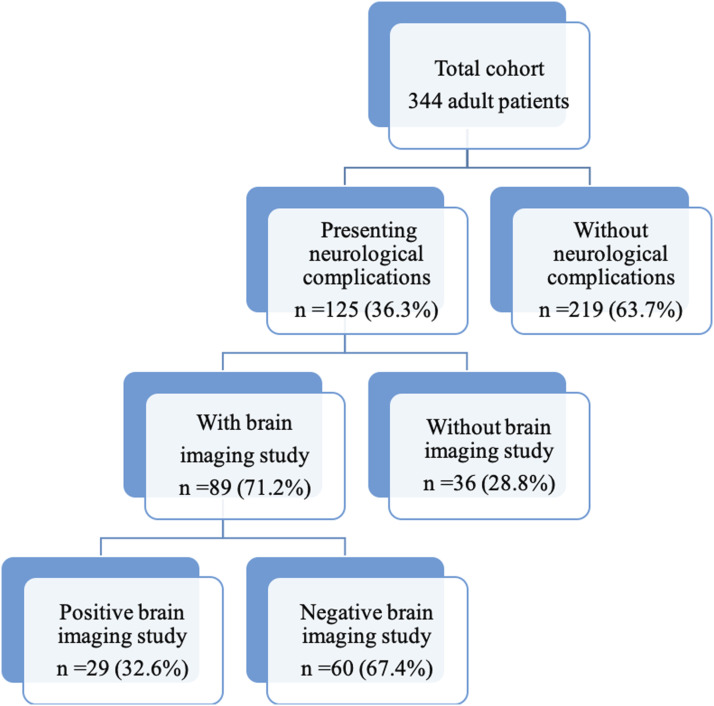
Total neurological events and brain imaging (CTs and MRIs) in the cohort of 344 patients.

### Spectrum of structural abnormalities on brain imaging

The clinical indications for brain imaging included focal neurologic signs (37.2% of patients with neurological symptoms), encephalopathy (34.0%), headache (12.4%), seizures (10.1%), trauma or fall (2.9%) and other causes including fever and sepsis (3.4%). In our cohort, the most frequent neurological final diagnosis in descending order were encephalopathy, polyneuropathy and strokes (Table 3). Table 4 shows positive findings in both CT and MRI scans performed in patients with neurological complications.

**Table 3.** Neurological complications and months post-transplant.

Complication	No. of events	Months post-transplant
Encephalopathy	45	1.4 (0–9)
Critical illness polyneuropathy	40	0.4 (0–4)
CVA (stroke)	17	0.78 (0–3)
Acute/subacute ischaemic infarct	16	
Subarachnoid haemorrhage	1	
Intraparenchymal haemorrhage	1 (also presented an acute ischaemic infarct)	
Headache	13	20.4 (4–36)
General seizures	9	1.9 (0.25–14)
Trauma	4	9.3 (3–26)
CNS malignancies	2	3.1 (1.5–4)
CNS infections	2	1.6 (0,13–3)
		Average time = 4.9
		**Parentheses show min and max

**Table 4.** Findings in CT and MRI scans.

Brain imaging studies in patients with neurological complications		
Neurological complication	CT	MRI
Positive findings/No. studies performed		
Encephalopathy	1/32 (3.12%)	3/5 (60%)
Stroke	19/22 (86.4%)	6/6 (100%)
Seizures	1/7	2/3
CNS malignancies	2/2	1/1
CNS infections	3/3	2/2
Headache	1/9	1/3

### Encephalopathy

**Figure 2.**
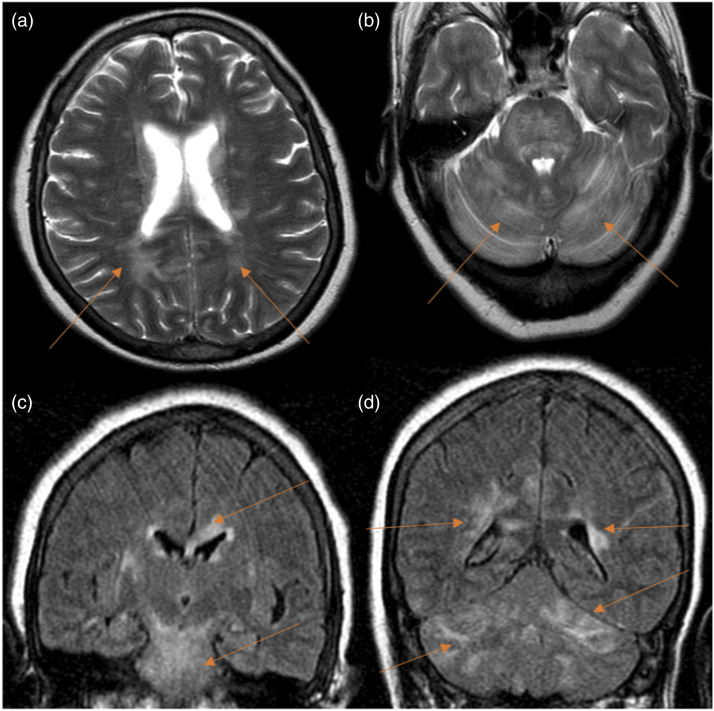
Encephalopathy case. A 63-year-old female patient with chronic obstructive pulmonary disease underwent double-LT. Seven days after the intervention, she presented with headache, distal tremor and temporospatial disorientation. CT scan did not reveal any abnormalities (not shown). Figures: axial MRI T2-weighted sequence (a and b) and coronal MRI FLAIR sequences (c and d). In the supratentorial region, there are scattered hyperintensities in deep-posterior white matter, corpus callosum and corona radiata (arrows in a, c and d). There are also hyperintensities (arrows in b, c and d) affecting the infratentorial region (white matter of cerebellum hemispheres, central midbrain and pons). These hyperintensities disappeared in the follow-up images. These findings were related to posterior reversible encephalopathy syndrome (PRES) with an atypical distribution due to drug toxicity (immunosuppressant drugs).

Alterations of consciousness and encephalopathy were common after transplantation, ranging from confusion and delirium to stupor and coma. Encephalopathy was the leading cause of neurological complication in our cohort. Forty-five patients suffered from this condition and 32 of them had brain imaging studies. Only one of the 28 CT studies revealed the underlying cause of encephalopathy (stroke). Nevertheless, 3 of the 5 MRIs performed showed abnormalities (Table 4). One of the positive MRI studies revealed scattered hyperintensities in the supratentorial region, affecting the deep posterior white matter, corpus callosum and corona radiata. There were also hyperintensities affecting the infratentorial region in the white matter of cerebellum hemispheres, central midbrain and pons. These findings were probably related to PRES with an atypical distribution due to drug toxicity (Figure 2). Drug toxicity due to tacrolimus was the main cause of encephalopathy in our cohort. Neurotoxicity of calcineurin inhibitors (manifested with altered consciousness, seizures and cortical blindness) was more common with higher intravenous dosing in the early postoperative period. In our cohort, the average time after LT to encephalopathy was 1.4 months.

### Cerebrovascular complications

**Figure 3.**
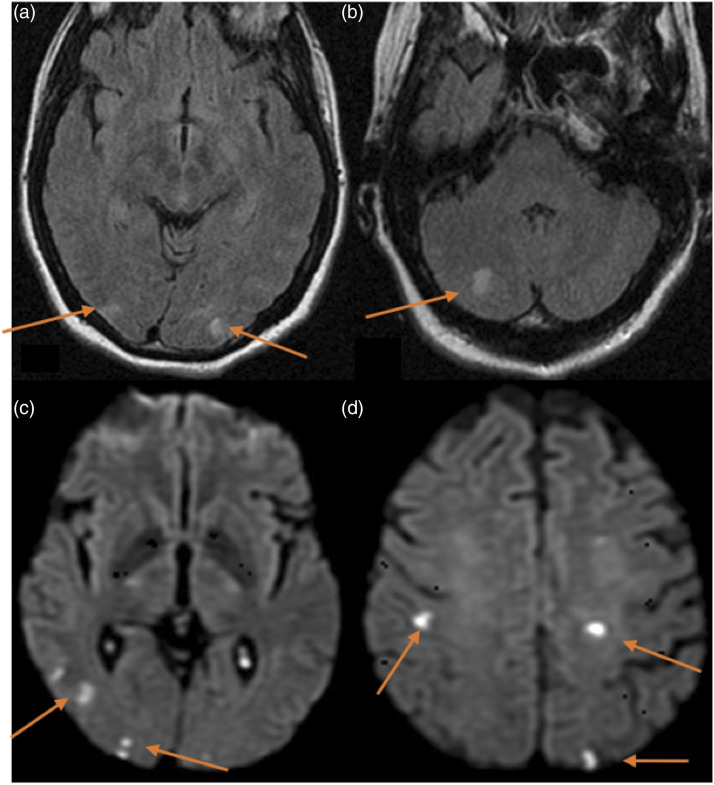
Multiple cerebral emboli case. A 58-year-old male patient with chronic obstructive pulmonary disease underwent double-LT. He presented an acute motor deficit in the right superior extremity 3 years after the transplantation. Besides, atrial fibrillation was recently diagnosed. Figures: axial MRI FLAIR sequences (a and b) and MRI diffusion-weighted sequences (c and d). Multiple cortical and subcortical hyperintense foci in occipito-parietal lobes (arrows in a and b). Some of these foci show restricted diffusion (arrows in c and d) related to acute infarctions.

**Figure 4.**
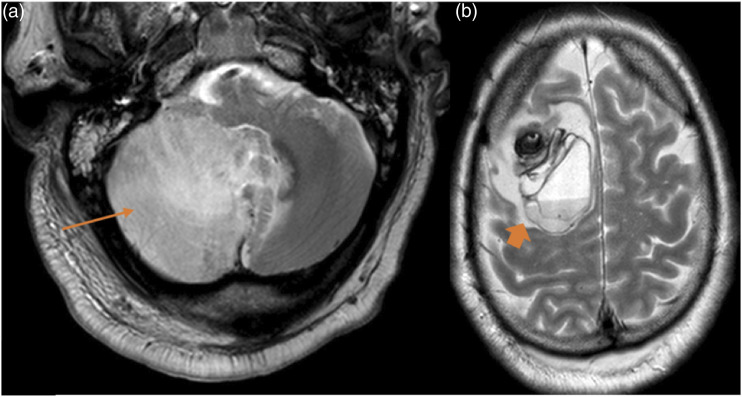
Ischaemic infarct and intraparenchymal haemorrhage. A 53-year-old male patient with usual interstitial pneumonia underwent double-LT. Five days after the transplantation, he presented a decreased level of consciousness. Figures: axial T2-weighted sequences (a and b). Right cerebellum infarction (thin arrow) with IV ventricle compression. Right frontal intraparenchymal haemorrhage (thick arrow) near to the derivation valve-shunt.

**Figure 5.**
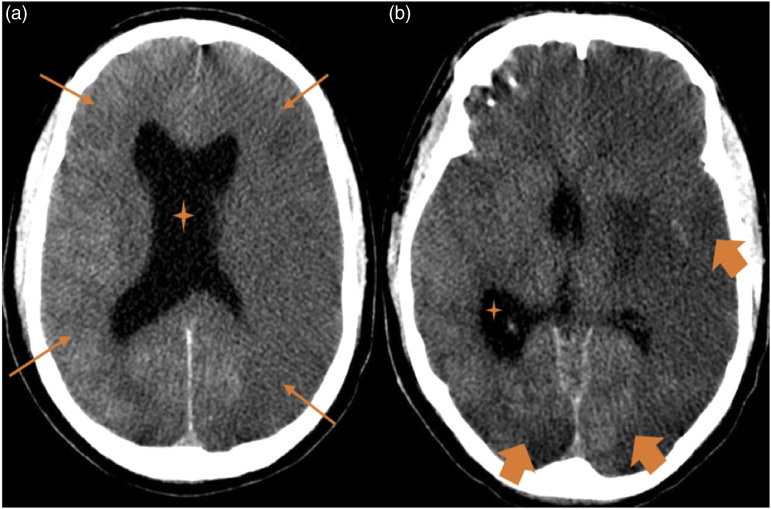
Fatal ischaemic stroke. A 23-year-old man patient with cystic fibrosis underwent double-LT and suffered two cardiac arrests during the intervention. After the transplantation, he presented non-reactive mydriasis and absence of brainstem reflexes. Figures: axial CT scan (a and b), diffuse cerebral oedema (thin arrows) and supratentorial hydrocephalus (stars). Left middle-cerebral artery and bilateral posterior-cerebral artery ischaemic strokes (thick arrows).

**Figure 6.**
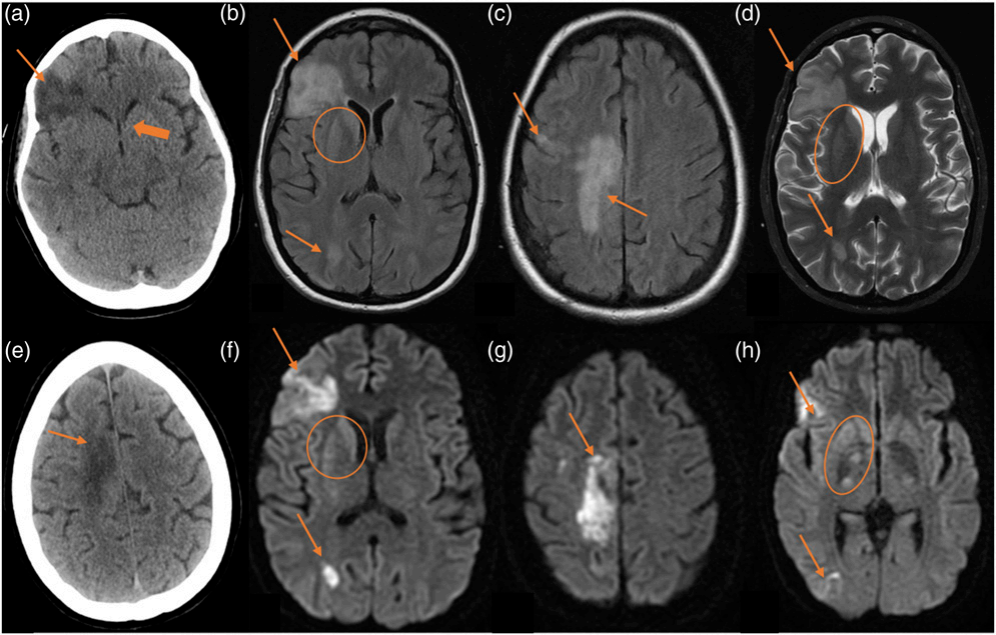
Multiple ischaemic strokes and anoxic encephalopathy. A 35-year-old woman with cystic fibrosis underwent double-LT. She suffered from severe haemodynamic compromise secondary to septic shock 5 days after the intervention. Figures: axial CT scan (a and e), axial MRI FLAIR sequence (b and c), axial MRI T2-weighted sequence (d) and axial MRI diffusion-weighted (f–h). CT scan reveals ischaemic strokes (arrows in a and b) and brain swelling with collapse of the ventricular system (thick arrow in a). There are large corticosubcortical hyperintensities on T2 and FLAIR (arrows in b–d), with restricted diffusion (arrows in f–h), in the right fronto-opercular, right parasagittal frontal and right occipito-parietal cortical regions. All of these hyperintensities are compatible with subacute ischaemic strokes. There are also hyperintensities on T2 and FLAIR with restricted diffusion (circles in b, d, f and h) in the right caudate and globus pallidus related to anoxic encephalopathy.

Cerebrovascular complications occurred in 17 patients and constitute the third most common neurological complication in our cohort. Thirteen patients had strokes in the first month after LT. Twenty-seven CT scans were performed in these patients: 24 CT scans showed cerebrovascular complications and 3 CT scans were negative. Seven MRI studies were performed and all of them revealed ischaemic lesions (Table 4). Eight patients had strokes within the first week of lung transplantation. There were two fatal cerebrovascular events (fatal ischaemic strokes), in which the duration from stroke to death was of 48 hours or less. The vascular events included six multiple cerebral emboli (Figure 3), three watershed/hypotensive ischaemic infarctions, two watershed/hypotensive ischaemic infarctions with multiple cerebral emboli, two basilar artery ischaemic strokes, one posterior cerebral artery ischaemic stroke with intracerebral haemorrhage (Figure 4), one middle cerebral artery ischaemic stroke (Figure 5), one subarachnoid haemorrhage and finally, one case of multiple ischaemic strokes and anoxic encephalopathy with restricted diffusion in right basal ganglia (Figure 6). Out of the 17 patients with cerebrovascular complications, 11 died. The average time after LT to cerebrovascular complication was 0.78 months.

### Seizures

**Figure 7.**
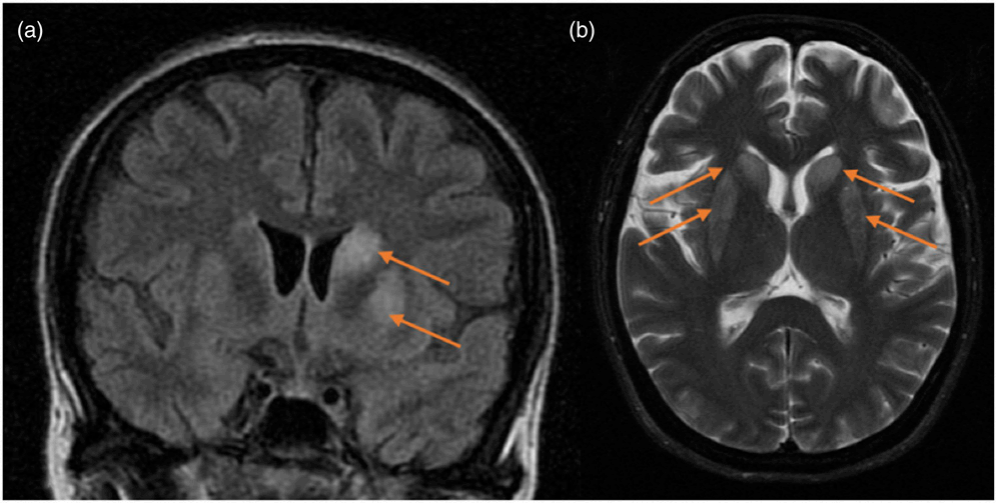
Diffuse hypoxic ischaemic encephalopathy. A 53-year-old female patient with primary pulmonary hypertension underwent double-LT. Figures: coronal MRI FLAIR sequence (a) and axial MRI T2-weighted sequence (b). The images show bilateral symmetrical hyperintensities and swelling in both caudate and putamina, left more than right (arrows).

Generalized seizures occurred in 9 patients. Most commonly, seizures presented as generalized tonic–clonic events with primary or secondary generalization. Imaging was normal in 8 patients and was informative in two patients (Figure 7). Only one of the 7 CT scans performed showed abnormalities (endophthalmitis case, explained in CNS infections) and one of the three MRI studies revealed signs of diffuse hypoxic-ischaemic encephalopathy with bilateral symmetrical hyperintensities in both caudate and putamina. (Table 4). The average time after LT to seizures was 1.9 months.

### CNS malignancies

**Figure 8.**
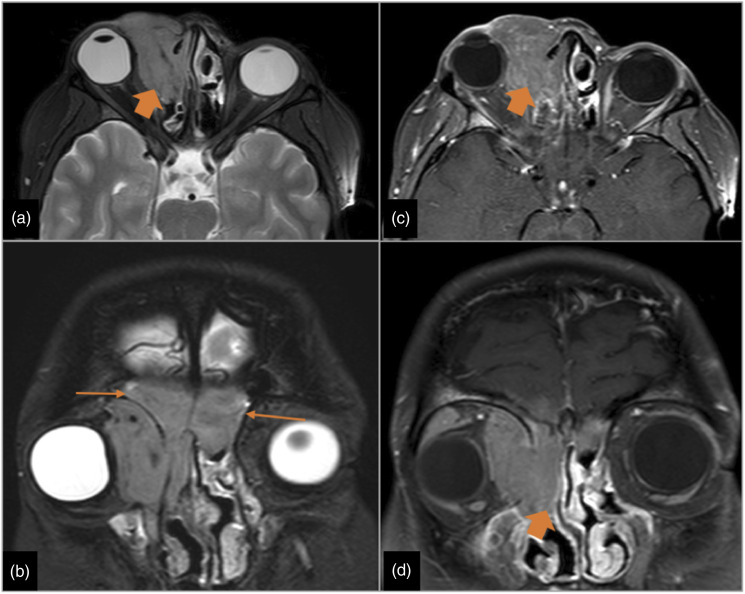
B-cell lymphoproliferative disorder. A 21-year-old woman with double-LT due to cystic fibrosis, started with right exophthalmos 5 months after the transplantation. Figures: axial MRI T2-weighted sequence (a), coronal MRI Stir sequence (b), axial MRI intravenous contrast T1-weighted sequence (c) and coronal MRI intravenous contrast T1-weighted sequence (d). A right orbital mass that displaces the eyeball (thick arrow in a and b) and reaches the right nasal cavity (thick arrow in c) and both frontal sinuses (thin arrows in d). The mass appears hyperintense and intermediate intensity on T2 sequences, hypointense on T1 sequences and shows moderate and uniform post contrast enhancement.

Two patients had CNS malignancies. A small meningioma was incidentally discovered in one male patient in the second month after the LT. A twenty-one-year-old female patient was diagnosed with B-cell lymphoproliferative disorder affecting the orbit (Figure 8). The haematologic diagnosis was an Epstein–Barr virus–associated lymphoproliferative disorder, in particular, a post-transplant–associated lymphoproliferative disorder, and it was discovered 4 months after the transplant. The average time after LT to CNS malignancies was 3.1 months.

### CNS infections

**Figure 9.**
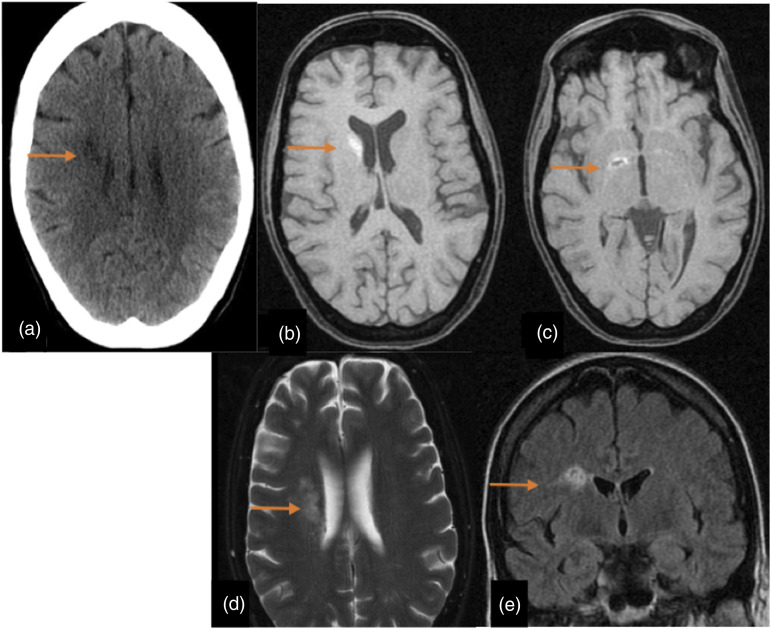
Cryptococcal CNS infection. A 41-year-old woman with primary pulmonary hypertension underwent double-LT. She was diagnosed with disseminated cryptococcal disease and the neuroimaging study was performed to rule out CNS infection. Figures: axial CT scan (a), axial T1-weighted sequences (b and c), axial T2-weighted sequences (d) and coronal FLAIR sequence (e). In CT, there are small hypodense lesions in right corona radiata (arrows).There are three hyperintense small polycystic lesions on the head of the right caudate nucleus, right internal capsule and right corona radiata (arrows).

**Figure 10.**
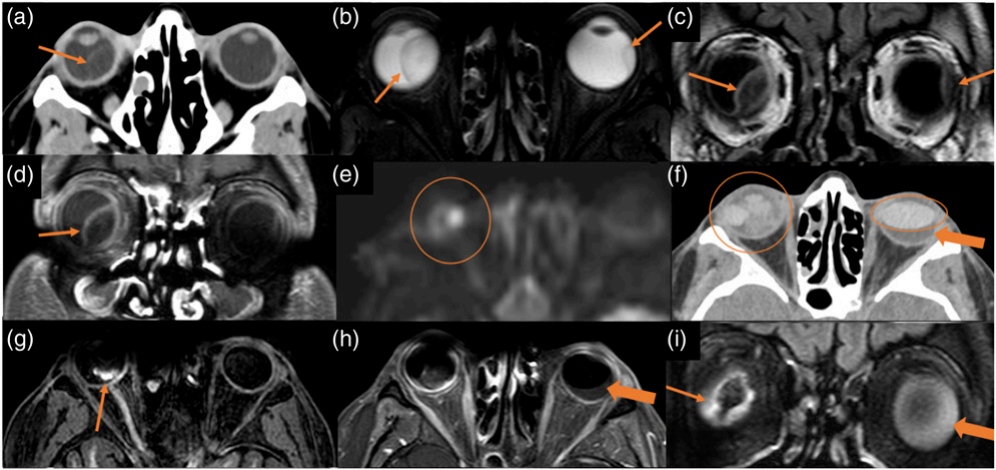
Choroidal abscesses and subretinal haemorrhage. 25-year-old woman with cystic fibrosis underwent double-LT. She had severe retro-ocular headache 4 days post-transplant and both CT and MRI scans were performed. Figures: axial CT scan (a), axial T2-weighted sequence (b), coronal FLAIR sequence (c), axial intravenously enhanced T1-weighted (d) and axial diffusion sequence (e). There are subretinal collections in both eyes (arrows in a–d), showing internal hyperintensity on T2 and FLAIR, with restricted diffusion, and peripheral enhancement. These findings are consistent with choroidal abscesses. During the follow-up, she underwent surgery with intraocular silicone placement. Figures: axial CT scan (f), non-contrast 3DT1 sequence (g), axial T1-weighted sequence (h) and coronal FLAIR sequence (i). Posterior to the left intraocular silicone, there is a crescent-shaped collection, hypodense on CT scan, hypointense on T1 and hyperintense on FLAIR, compatible with subretinal haemorrhage. There is poor visualization of the right ocular globe due to magnetic susceptibility artefact. The left intraocular silicone is irregular and has moved anteriorly. There is posterior right chamber occupation with high-density areas on the CT scan and hyperintense areas on T1, without evidence of enhancement, compatible with subretinal haemorrhages.

CNS infection was recorded in two cases. One female patient with disseminated cryptococcal disease had imaging evidence of CNS infection (Figure 9). Three months post-transplant, CT scans showed small hypodense lesions in right corona radiata and MRI scans revealed three hyperintense small polycystic lesions on the head of the right caudate nucleus, right internal capsule and right corona radiata. The second case was a young female patient with several postoperative complications such as mediastinitis, bacteraemia and panuveitis. She suffered severe retro-ocular headache 4 days post-transplant and both the CT as the MRI scans demonstrated bilateral choroidal abscesses (Figure 10). During the follow-up, she underwent surgery with intraocular silicone placement. During the postoperative period, she presented seizures and the MRI scan performed showed bilateral subretinal haemorrhage and PRES.

### Others

Severe neuromuscular complications due to severe critical illness polyneuropathy occurred in 40 patients and constitute the second most common neurological complication in our cohort. These patients were exposed to intravenous corticosteroids and some of them to multiple organ failure and sepsis. We observed 4 patients suffering from postoperative entrapment neuropathies related to traction and stretch injury (upper and lower trunk brachial plexopathy). Headache was present in 13 patients. Only in one case (endophthalmitis), the neuroimaging studies revealed the cause. Two of those patients presented severe headache as an early manifestation of neurotoxicity due to the calcineurin inhibitor (tacrolimus). Post-traumatic haemorrhage events related to accidents were found in 4 patients: 1 subdural haematoma, 1 epidural haematoma, 1 intracerebral haemorrhage with subarachnoid and intraventricular haemorrhage, and 1 subarachnoid haemorrhage.

## Discussion

Lung transplantation (LT) constitutes a potentially life-saving procedure for many patients, and every year, the number of lung recipients continues to increase. Since the neurological complications after LT are common,^2,3,5,6^ our purpose was to analyse the neuroimaging findings of a cohort of adult patients who underwent lung transplantation. The influence of nervous system disorders on lung transplant recipients and their survival has been studied by some articles. Mateen et al. described that neurological complications in LT were related to increased risk of death.^5^ Gamez et al. did not find associations with mortality.^2^ They observed that neurological complications affected the mean length of hospital stay and especially the time spent in the intensive care unit. Smith et al. reported that the 36%–44% of the patients exhibited postoperative delirium, which was associated with worse short-term clinical outcomes.^3^ They found neurological sequelae were common, occurring in 45% of patients.^3^ In other organ transplant recipients, neurological complications have a significant effect on both morbidity and mortality.^6–15^

On the other hand, our review focused on neuroimaging findings as not many articles have been previously published about this theme. Besides, we have studied the largest LT cohort in the literature. Brain imaging detected structural abnormalities in our study in one-third of patients (32, 6%) who had CT or MR studies performed. Since two of the most common neurological complications in our study were encephalopathy and strokes, the role of the radiologist is crucial to identify factors that inform or have an impact on the acute stabilization and management of the patient. Structural abnormalities on brain imaging were detected only on the MRI studies (with CT scans negatives) for indications of encephalopathy and seizures, and in three cases of strokes (Table 4). This finding is consistent with the results of other studies, where MRI was superior to CT and mandatory for the diagnosis of structural abnormalities in patients whose clinical indications for imaging evaluation were encephalopathy, confusion, seizures or focal neurological signs. In our study, the frequency of clinical neurological events was 36.3%, lower than those reported in the available studies.^2,3,5,16,17^ One reason for this difference is probably related to the surveillance period in a retrospective study. The data collection was made between 2018 and 2020 so patients who underwent LT in the last years were not followed for a long time (available LT series had a surveillance period of 10 years). Encephalopathy, critical illness polyneuropathy and stroke, in that order, were the most frequent CNS complications. Our findings are similar to those reported in other adult series.^2,3,5^ For instance, Mateen et al. found that the most common neurological complications in the CNS were encephalopathy, strokes and seizures.^5^ Smith et al. published the most common early neurological sequelae were postoperative delirium and posterior reversible encephalopathy syndrome, followed by stroke/transient ischaemic attack and neurotoxicity.^3^ In our cohort, the average time after LT to a neurological complication was 4.9 months post-transplant. We observed the shortest time in the group of patients suffering from strokes (0.78 months). In other studies, the neurological complications also appeared in the first year post-transplant.^2,3,5^ One of the most important limitations in our study was the reduced use of MRI scans in our cohort. Approximately four times more CT scans than MRI scans were performed (99 CTs compared to 22 MRIs). For instance, there were 45 patients with encephalopathy and just 5 of them had an MRI study. We attribute this variation to the fact that many of these patients were critically ill. Brain CT was often the initial investigation in these patients because of its availability, rapidity, patient tolerance and sensitivity to acute neurosurgical emergencies. Since the MRI studies have proven to be the best imaging tool among these patients, it seems we could have lost important information in our cohort. This is one of the main problems of a retrospective study where there had been no established MRI protocols for neurological complications. There are other limitations in our retrospective study. Our study design may not have identified all CNS complications since minor complications that do not generally require brain imaging would not have been captured in this study. Future studies in our cohort can investigate the relationship between positive neurological brain imaging and survival or mortality rates.

In conclusion, LT recipients constitute an especially vulnerable group that needs a close surveillance, mainly during the early post-transplant period. We studied the largest LT cohort in literature and encephalopathy, critical illness polyneuropathy and stroke, in that order, were the most frequent CNS complications.

CT is a very useful method to evaluate the vascular complications in the early period after transplantation and could be considered the first imaging technique for stroke cases. However, MRI proved to be the best imaging tool among all these patients but especially in patients with encephalopathy and seizures and should always be performed as the imaging technique of choice if possible.
